# AI-Assisted Hypothesis Generation to Address Challenges in Cardiotoxicity Research: Simulation Study Using ChatGPT With GPT-4o

**DOI:** 10.2196/66161

**Published:** 2025-05-15

**Authors:** Yilan Li, Tianshu Gu, Chengyuan Yang, Minghui Li, Congyi Wang, Lan Yao, Weikuan Gu, DianJun Sun

**Affiliations:** 1 The Second Affiliated Hospital of Harbin Medical University Harbin China; 2 Department of Clinical Pharmacy and Translational Science, University of Tennessee Health Science Center Memphis, TN United States; 3 Department of Orthopedic Surgery and BME-Campbell Clinic University of Tennessee Health Science Centre Memphis, TN United States; 4 Diabetes Research Center Qatar Biomedical Research Institute (QBRI) Hamad Bin Khalifa University (HBKU) Doha Qatar; 5 College of Health Management Harbin Medical University Harbin China; 6 Lt. Col. Luke Weathers, Jr. VA Medical Center Memphis, TN United States; 7 Department of Pharmaceutical Sciences University of Tennessee Health Science Center Memphis, TN United States; 8 The Second Affiliated Hospital of Harbin Medical University Centre for Endemic Disease Control, Chinese Centre for Disease Control and Prevention, Harbin Medical University Key Laboratory of Etiologic Epidemiology, Education Bureau of Heilongjiang Province & Ministry of Health Harbin China

**Keywords:** cardiotoxicity, ChatGPT with GPT-4o, artificial intelligence, AI, heart, hypothesis generation

## Abstract

**Background:**

Cardiotoxicity is a major concern in heart disease research because it can lead to severe cardiac damage, including heart failure and arrhythmias.

**Objective:**

This study aimed to explore the ability of ChatGPT with GPT-4o to generate innovative research hypotheses to address 5 major challenges in cardiotoxicity research: the complexity of mechanisms, variability among patients, the lack of detection sensitivity, the lack of reliable biomarkers, and the limitations of animal models.

**Methods:**

ChatGPT with GPT-4o was used to generate multiple hypotheses for each of the 5 challenges. These hypotheses were then independently evaluated by 3 experts for novelty and feasibility. ChatGPT with GPT-4o subsequently selected the most promising hypothesis from each category and provided detailed experimental plans, including background, rationale, experimental design, expected outcomes, potential pitfalls, and alternative approaches.

**Results:**

ChatGPT with GPT-4o generated 96 hypotheses, of which 13 (14%) were rated as highly novel and 62 (65%) as moderately novel. The average group score of 3.85 indicated a strong level of innovation in these hypotheses. Literature searching identified at least 1 relevant publication for 28 (29%) of the 96 hypotheses. The selected hypotheses included using single-cell RNA sequencing to understand cellular heterogeneity, integrating artificial intelligence with genetic profiles for personalized cardiotoxicity risk prediction, applying machine learning to electrocardiogram data for enhanced detection sensitivity, using multi-omics approaches for biomarker discovery, and developing 3D bioprinted heart tissues to overcome the limitations of animal models. Our group’s evaluation of the 30 dimensions of the experimental plans for the 5 hypotheses selected by ChatGPT with GPT-4o revealed consistent strengths in the background, rationale, and alternative approaches, with most of the hypotheses (20/30, 67%) receiving scores of ≥4 in these areas. While the hypotheses were generally well received, the experimental designs were often deemed overly ambitious, highlighting the need for more practical considerations.

**Conclusions:**

Our study demonstrates that ChatGPT with GPT-4o can generate innovative and potentially impactful hypotheses for overcoming critical challenges in cardiotoxicity research. These findings suggest that artificial intelligence–assisted hypothesis generation could play a crucial role in advancing the field of cardiotoxicity, leading to more accurate predictions, earlier detection, and better patient outcomes.

## Introduction

### Background

Cardiotoxicity is critically important in heart disease research. Cardiotoxicity can lead to severe and sometimes irreversible damage to the heart muscle, resulting in conditions such as heart failure, arrhythmias, and even death [1,2]. For patients with preexisting heart conditions, it is essential to understand how new treatments might impact their cardiovascular health. Understanding and mitigating cardiotoxicity is crucial for patient safety, especially for those undergoing treatment for other conditions such as cancer, where chemotherapy drugs are known to have cardiotoxic effects [3-7].

Current studies on cardiotoxicity focus on understanding the underlying mechanisms, early detection, and the prevention of cardiac damage caused by various treatments, particularly chemotherapy and other drugs [8,9]. Researchers are investigating biomarkers, genetic factors, and advanced imaging techniques to better predict and monitor cardiotoxic effects [8-12]. However, the research faces significant challenges, including the complexity of cardiotoxic mechanisms, variability among patients, the limitations of animal models, and difficulties in early detection and long-term monitoring [6,13,14]. In addition, ethical and practical constraints in human studies, heterogeneous data sources, and technological limitations further complicate efforts to develop effective strategies for managing cardiotoxicity [13-16].

The application of artificial intelligence (AI) in drug toxicity is increasingly important due to its potential to revolutionize how we predict, detect, and mitigate the adverse effects of drugs, including cardiotoxicity [17-21]. AI algorithms can analyze vast datasets from preclinical studies, clinical trials, and real-world evidence to identify patterns and predict toxicity risks with greater accuracy and speed than traditional methods [22,23]. Current progress includes the development of machine learning models that can forecast toxicity based on chemical structure and biological activity and AI-driven platforms that integrate multi-omics data to provide comprehensive toxicity profiles [22,23]. These advancements not only enhance drug safety and efficacy but also streamline the drug development process, reduce costs, and improve patient outcomes by enabling more personalized treatment strategies [11,12,24].

The integration of AI models such as ChatGPT with GPT-4o into hypothesis generation presents novel ethical and academic challenges that must be carefully addressed. While the use of AI has the potential to accelerate scientific discovery by generating innovative research questions and experimental designs, it also raises concerns regarding scientific attribution, research integrity, and the possible misuse of AI-generated content.

### Objectives

While studies have demonstrated the ability of ChatGPT with GPT-4o to propose and evaluate hypotheses in theoretical domains [25,26], the systematic assessment of AI-generated hypotheses in the biomedical field, particularly in cardiotoxicity research, has not been reported. Unlike mathematical or purely theoretical investigations, biomedical hypothesis testing requires experimental validation, which introduces additional challenges such as feasibility, clinical relevance, and ethical considerations.

Accordingly, this study investigates whether ChatGPT with GPT-4o can generate hypotheses to address challenges in cardiotoxicity research and whether the hypotheses are novel and potentially able to impact research and clinical applications in heart disease treatment.

## Methods

### Hypothesis Generation

We investigated the ability of ChatGPT with GPT-4o to generate hypotheses to overcome 5 major challenges in cardiotoxicity research in 5 separate sessions, each targeting 1 of the 5 challenges (shown in Textbox 1) in cardiotoxicity research. In each session, we used a structured, role-based approach. ChatGPT with GPT-4o was instructed to act as a biomedical research scientist and was prompted as follows: “Please propose as many innovative and feasible scientific hypotheses as possible that address the challenge of [eg, the complexity of mechanisms] in cardiotoxicity research. Each hypothesis should be concise, biologically plausible, and amenable to experimental validation.” We avoided imposing strict length or keyword constraints to preserve creative diversity in the responses. Each session generated between 18 and 22 initial hypotheses. The breakdown per category is also shown in Textbox 1.

**Five major challenges in cardiotoxicity research, with the number of hypotheses per challenge shown in parentheses.**

1. Complexity of mechanisms (16 hypotheses): cardiotoxicity can result from various mechanisms, including oxidative stress, inflammation, apoptosis, and autophagy. Understanding these complex biological processes and how they interact is challenging.
2. Variability among patients (20 hypotheses): patients exhibit different levels of susceptibility to cardiotoxicity due to genetic, environmental, and lifestyle factors. This variability makes it difficult to predict cardiotoxicity and develop universal guidelines.
3. Detection sensitivity (20 hypotheses): the early detection of cardiotoxicity is crucial but challenging. Traditional diagnostic methods, such as echocardiography or biomarkers, may not detect subtle early changes, delaying intervention and increasing the risk of severe damage.
4. Biomarker identification (20 hypotheses): identifying reliable biomarkers for cardiotoxicity is essential for early detection and monitoring but remains a significant challenge. Biomarkers need to be sensitive, specific, and validated in diverse patient populations.
5. Limitations of animal models (20 hypotheses): while animal models are essential for preclinical studies, they do not always accurately replicate human cardiotoxicity. Differences in heart physiology and drug metabolism between animals and humans can lead to discrepancies in results.

### Experimental Plan Generation

Our investigation involved 2 steps [27]:

1. We asked ChatGPT with GPT-4o to generate as many hypotheses as possible to address 5 major challenges in cardiotoxicity research.
2. We asked ChatGPT with GPT-4o to select the best hypothesis from each of the 5 groups of hypotheses corresponding to the 5 challenges based on a 5-point scale, considering aspects such as innovation, relevance to cardiotoxicity research, and practical applicability (the evaluation criteria are presented in
  Multimedia Appendix 1
  ). For each selected hypothesis, we asked for a detailed investigation plan, including background, rationale, experimental design to test the hypothesis, expected outcomes from the investigation, potential pitfalls, and alternative approaches to overcome the pitfalls.

For both steps, we checked the novelty and feasibility of the hypotheses and compared their respective advantages and disadvantages. Each experimental plan was also evaluated. Three evaluators independently reviewed the hypotheses and each dimension of the experimental plans. The evaluators were YL, a professor holding both MD and PhD degrees; TG, an MD degree holder and final-year PhD student; and CY, an MD degree holder and first-year PhD student. Final scores for each hypothesis and experimental plan were determined through group discussion conducted via web-based meetings. Each hypothesis was independently scored by the 3 evaluators before the group discussion. Fleiss κ was used to assess interrater reliability. In cases of disagreement, the scores were discussed, and a consensus score was reached through group discussion.

The hypotheses were evaluated based on their novelty and relevance in the context of existing research (the evaluation criteria are presented in Multimedia Appendix 1). The scoring rubric was as follows: 5=novel hypothesis, 4=relatively novel hypothesis, 3=slightly novel hypothesis, 2=low novelty, and 1=not novel. For the detailed experimental plans, the evaluators applied grading scales based on the detailed information provided by ChatGPT with GPT-4o (the individual sets of hypotheses are presented in Multimedia Appendix 1). The evaluations were conducted over a 2-week period beginning on August 12, 2024.

Our expert panel accounted for differences across scientific subfields. In addition to experts in heart diseases (YL, CW, and DS), the panel included experts in statistics (LY and ML) and genomics (WG). Publications were identified using keywords derived from the hypotheses, and the experts read these publications and discussed them within the group to achieve a nuanced understanding of scientific innovation.

### Evaluation Procedure

Novelty was evaluated by the 3 independent investigators using a structured 5-point scale, similar to methodologies used in previous studies assessing AI-generated content and scientific hypothesis evaluation [28,29]. To ensure scoring consistency, we calculated interrater reliability using Cohen κ for pairwise comparisons and Fleiss κ for overall agreement across multiple raters. A κ value of >0.75 was considered strong agreement, while values between 0.40 and 0.75 indicated moderate agreement. In addition, beyond novelty and feasibility, we introduced reliability as an evaluation criterion. This involved assessing the following aspects: supporting evidence, which considers whether existing literature provides a foundation for the hypothesis; and plausibility, which considers whether the proposed mechanisms align with established scientific knowledge. Each hypothesis was assigned a score (ranging from 1 to 5) for reliability, following the same rating scale used for novelty and feasibility.

Thus, each evaluator scored the hypotheses using a structured 5-point scale based on 3 criteria: novelty, feasibility, and reliability. The scoring rubric was as follows: 5=highly novel, feasible, and reliable (represents a significant departure from existing literature; strong foundation; testable with current methods); 4=moderately novel, feasible, and reliable (builds on prior work with a novel angle or methodology; may require some technical adaptation); 3=slightly novel, feasible, and reliable (modest innovation or adjustment of existing theories; some limitations); 2=low novelty, feasibility, and reliability (minor variations on well-known ideas; substantial experimental or logical constraints); and 1=not novel, feasible, and reliable (direct replication of existing studies; impractical or unsupported by evidence; Table 1).

**Table 1:** Evaluation rubric for artificial intelligence–generated hypotheses across 3 criteria (novelty, feasibility, and reliability).

Score	Novelty	Feasibility	Reliability
5	Highly novel: represents a significant departure from existing literature	Highly feasible: testable with current experimental methods and technologies	Highly reliable: strong supporting evidence and alignment with established scientific understanding
4	Moderately novel: builds on prior work with a new angle or method	Moderately feasible: requires some technical or methodological adaptation	Moderately reliable: some literature support; plausible but needs validation
3	Slightly novel: modest innovation; modification of known ideas	Feasible with limitations: presents notable challenges but testable with adjustments	Somewhat reliable: theoretical plausibility with limited evidence
2	Low novelty: minor variation on existing concepts	Limited feasibility: significant technical and logistical constraints	Low reliability: sparse or questionable supporting evidence
1	Not novel: replicates known work without new perspective	Not feasible: impractical with current methods; lacks clear experimental pathway	Not reliable: no credible support or conflicting with established knowledge

### Literature Search and Publication Count

To assess the novelty of each hypothesis, we conducted a structured PubMed search on August 12, 2024. For each hypothesis, 2 to 4 representative keywords were identified based on biologically relevant terms and core concepts in the hypothesis text. These keywords were independently reviewed by 2 evaluators (TG and CY). In cases of disagreement, a third senior reviewer (YL) facilitated discussion until consensus was reached. From the selected keywords, a Boolean search query (typically combining 2-3 keywords) was constructed for each hypothesis to retrieve relevant publications. The number of publications returned for each query was recorded and used as a proxy for the novelty of the hypothesis. Hypotheses associated with zero or very few publications (n=1-2) were considered more novel. In addition to publication count, at least 1 evaluator independently reviewed the retrieved articles to assess conceptual similarity and determine whether the hypothesis represented a novel direction, a variation on existing work, or a well-established idea. This approach provided both quantitative and qualitative evaluations of scientific originality.

### Ethical Considerations

This study did not involve the collection or use of human participant data, patient records, or identifiable personal information. Therefore, ethics committee approval and informed consent were not required. The hypotheses were generated by an AI model (ChatGPT with GPT-4o) and subsequently evaluated by expert reviewers based on publicly available scientific knowledge and literature. No sensitive or private human participant data were analyzed or accessed in this process.

## Results

### Hypotheses to Address Challenges in Cardiotoxicity Research

#### Overview

**Figure 1:**
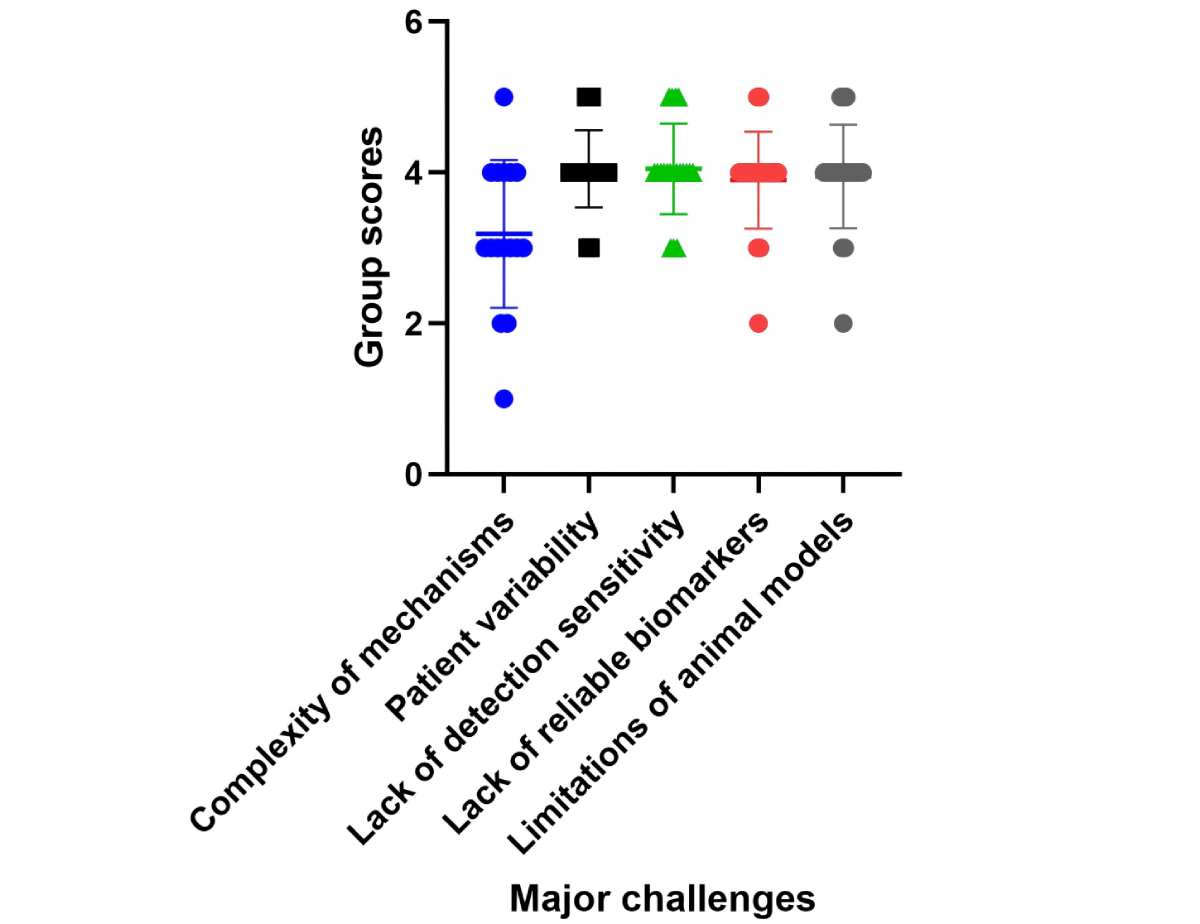
Distribution of evaluators’ group scores for artificial intelligence–generated hypotheses across 5 major challenges in cardiotoxicity research.

The 5 sets of 96 hypotheses addressed 5 challenges in cardiotoxicity research: the complexity of mechanisms, variability among patients, the lack of detection sensitivity, the lack of reliable biomarkers, and the limitations of animal models (the individual sets of hypotheses are presented in Multimedia Appendix 1). The 96 hypotheses were independently evaluated by 3 investigators and discussed by our group (Figure 1). The evaluations indicated a high level of innovation: of the 96 hypotheses, 13 (14%) were rated as highly novel (score of 5) and 62 (65%) as moderately novel (score of 4), while 16 (17%) were considered slightly novel (score of 3). A small portion (4/96, 4%) were rated as low novelty (score of 2). Only 1 (1%) of the 96 hypotheses was graded as not novel (score of 1). The average group score of 3.85 indicates a strong level of innovation, with many hypotheses (75/96, 78%) scoring between 4 and 5, particularly those leveraging advanced technologies such as AI, multi-omics, CRISPR (clustered regularly interspaced short palindromic repeats), and 3D bioprinting. The overall average score was approximately 4.0, suggesting a generally high level of novelty and potential for these hypotheses to advance cardiotoxicity research. While some areas, such as improving detection sensitivity and developing human-relevant models, received higher recognition, others, particularly those focused on metabolomics and existing technologies, were considered less novel.

These hypotheses share similarities in leveraging advanced technologies such as AI, multi-omics, CRISPR, 3D bioprinting, and organ-on-a-chip systems to improve the accuracy and relevance of cardiotoxicity research. They differ in their specific focus: understanding cellular mechanisms (eg, single-cell RNA sequencing [scRNA-seq]), personalizing risk prediction (eg, AI with genetic profiles), enhancing detection sensitivity (eg, machine learning on electrocardiogram [ECG] data), discovering biomarkers (eg, multi-omics integration), and creating human-relevant models (eg, 3D bioprinted heart tissues).

Literature searching identified at least 1 relevant publication for 28 (29%) of the 96 hypotheses. The number of related publications varied notably, ranging from 0 (0/16, 0% hypotheses) to 7125 for a hypothesis concerning scRNA-seq (Table 2).

**Table 2:** Evaluation of hypotheses to overcome the challenge of the complexity of mechanisms in cardiotoxicity research.

Hypotheses	Novelty	Keywords	Publications (n=19,471), n (%)	Evaluator 1^a^ score	Evaluator 2^b^ score	Evaluator 3^c^ score	Group consensus score
1. Machine learning models can predict cardiotoxicity by integrating multi-omics data	Integration of advanced AI^d^ with multi-omics for toxicity prediction	“machine learning,” “multi-omics,” “toxicity prediction”	0 (0)	4	4	4	4
2. CRISPR^e^-Cas9 gene-editing technology can create precise cellular models mimicking human cardiotoxicity	Use of CRISPR-Cas9 for precise human-like cellular models	“CRISPR-Cas9,” “cellular models,” “cardiotoxicity”	10 (0.05)	4	4	3	4
3. High-throughput screening using iPSC^f^-derived cardiomyocytes can identify novel biomarkers of cardiotoxicity	Application of iPSC-derived cardiomyocytes in high-throughput screening	“iPSC,” “high-throughput screening,” “biomarkers”	57 (0.29)	3	3	3	3
4. Single-cell RNA sequencing can dissect heterogeneous cellular responses to cardiotoxic agents	Using single-cell RNA sequencing for detailed cellular response analysis	“single-cell RNA sequencing,” “cellular response”	7125 (36.59)	3	1	4	2
5. Advanced imaging techniques like super-resolution microscopy can visualize real-time cardiomyocyte responses	Real-time visualization of cellular responses with super-resolution microscopy	“super-resolution microscopy,” “real-time visualization”	60 (0.31)	3	3	3	3
6. Systems biology approaches can map out molecular interactions leading to cardiotoxicity	Comprehensive systems biology approach for mapping molecular interactions	“systems biology,” “molecular interactions,” “cardiotoxicity”	9 (0.05)	4	4	3	4
7. Organoid models of human heart tissue can study cardiotoxicity in a physiologically relevant context	Use of organoid models for physiologically relevant cardiotoxicity studies	“organoid models,” “human heart tissue,” “cardiotoxicity”	22 (0.11)	3	3	3	3
8. AI integrated with pharmacokinetic and pharmacodynamic data can predict individual susceptibility to cardiotoxicity	Combining AI with pharmacokinetic data for personalized susceptibility prediction	“AI,” “pharmacokinetics,” “susceptibility prediction”	102 (0.52)	3	3	3	3
9. Epigenetic profiling can reveal how cardiotoxic agents alter DNA methylation and histone modifications	Epigenetic profiling for understanding DNA and histone modification changes	“epigenetics,” “DNA methylation,” “histone modification”	9703 (49.83)	3	1	4	3
10. Bioprinted 3D heart tissues can provide accurate models for studying cardiotoxicity	Accurate bioprinted 3D heart tissue models for cardiotoxicity research	“bioprinted 3D tissues,” “cardiotoxicity models”	5 (0.02)	4	4	4	4
11. Exploring the gut-heart axis can uncover how gut microbiota influence cardiomyocyte health	Exploring gut-heart microbiota interactions in cardiotoxicity	“gut-heart axis,” “microbiota,” “cardiomyocyte health”	2 (0.01)	5	5	4	5
12. Combining metabolomics with lipidomics can provide a comprehensive understanding of disrupted cellular metabolism	Integration of metabolomics and lipidomics for metabolic disruption analysis	“metabolomics,” “lipidomics,” “cellular metabolism”	1674 (8.6)	3	1	3	2
13. Investigating the influence of circadian rhythms on cardiotoxicity can reveal time-dependent variations in heart susceptibility	Studying time-dependent variations with circadian rhythm influences	“circadian rhythms,” “time-dependent variations”	221 (1.13)	3	2	3	3
14. Developing dual-target drugs can reduce cardiac damage while maintaining treatment efficacy	Development of dual-target drugs for reducing cardiac damage	“dual-target drugs,” “cardiac damage reduction”	0 (0)	4	4	3	4
15. Studying the interplay between oxidative stress and autophagy can identify novel therapeutic approaches	Understanding oxidative stress and autophagy interplay for therapeutic strategies	“oxidative stress,” “autophagy,” “therapeutic approaches”	481 (2.47)	3	2	1	1
16. Integrating real-time data from wearable devices can monitor cardiotoxicity and adapt treatments dynamically	Using wearable devices for real-time cardiotoxicity monitoring	“wearable devices,” “real-time monitoring,” “cardiotoxicity”	0 (0)	4	4	2	3

^a^Author YL (MD and PhD, professor).

^b^Author TG (MD, final-year PhD candidate).

^c^Author CY (MD, first-year PhD student).

^d^AI: artificial intelligence.

^e^CRiSPR: clustered regularly interspaced short palindromic repeats.

^f^iPSC: induced pluripotent stem cell.

#### Complexity of Mechanisms 

The 16 hypotheses proposed to overcome the challenge of the complexity of mechanisms in cardiotoxicity research emphasize the integration of advanced technologies and multidisciplinary approaches (more details are provided in the “Hypotheses to overcome the challenge of the complexity of mechanisms in cardiotoxicity research” section in Multimedia Appendix 1). These include using machine learning models with multi-omics data, CRISPR-Cas9 gene editing, induced pluripotent stem cell–derived cardiomyocytes for high-throughput screening, scRNA-seq, and super-resolution microscopy. Additional methods highlighted are systems biology approaches, organoid models, AI-integrated pharmacokinetic data, epigenetic profiling, and bioprinted 3D heart tissues. Other proposed directions include exploring the gut-heart axis, metabolomics, and circadian rhythms. The novelty of these hypotheses lies in their innovative application of cutting-edge technologies and comprehensive approaches to understanding and mitigating cardiotoxicity. Their significance is underscored by the potential to uncover intricate molecular interactions, identify novel biomarkers, and develop precise predictive models. The impact on cardiotoxicity research is substantial, promising advancements in early detection, personalized treatment, and the development of safer therapeutics, ultimately improving patient outcomes and reducing adverse cardiac effects.

The literature search was conducted on August 12, 2024, on PubMed. Publications were found for 13 (81%) of the 16 hypotheses. The number of related publications varied significantly, ranging from 0 to 7125 for a hypothesis concerning scRNA-seq (Table 2).

The evaluations rated 1 (6%) of the 16 hypotheses as highly novel (score of 5), 5 (31%) as moderately novel (score of 4), 7 (44%) as moderately innovative but less novel (score of 3), 2 (12%) as low novelty (score of 2), and 1 (6%) as not novel (score of 1). The highest group score of 5 was awarded to the hypothesis exploring the influence of the gut-heart axis on cardiomyocyte health. Other highly rated hypotheses (score of 4) included those focused on machine learning models with multi-omics data, CRISPR-Cas9 for creating cellular models, and bioprinted 3D heart tissues. The average score of 3.19 (SD 0.98) for these hypotheses suggests a strong emphasis on integrating advanced technologies such as machine learning, CRISPR, and multi-omics to address the complexity of cardiotoxicity mechanisms.

For the 16 hypotheses addressing the challenge of the complexity of mechanisms (Table 2), the group scores were as follows: 4, 4, 3, 2, 3, 4, 3, 3, 3, 4, 5, 2, 3, 4, 1, and 3. The average group score was calculated as 3.19 (SD 0.98; IQR 3.00-4.00). To assess consistency among the 3 evaluators (YL, TG, and CY), we computed Fleiss κ for the 16 hypotheses. On the basis of the raw ratings, the overall Fleiss κ was estimated to be 0.27, which indicates fair agreement among evaluators according to established guidelines.

#### Variability Among Patients 

The 20 hypotheses proposed to address the challenge of variability among patients in cardiotoxicity research introduce novel approaches that leverage advanced technologies such as AI, patient-specific genetic profiling, high-throughput screening with patient-derived cardiomyocytes, wearable health monitors, and multi-omics (more details are provided under “Hypotheses to overcome the challenge of variability among patients in cardiotoxicity research” in Multimedia Appendix 1). These hypotheses aim to predict individual susceptibility, identify genetic and epigenetic markers, uncover gene-drug interactions, and explore environmental and hormonal influences on cardiotoxicity. The significance lies in their potential to enable personalized medicine, improving drug safety and efficacy by tailoring treatments to individual patient profiles. The impact on cardiotoxicity research includes the development of predictive biomarkers, noninvasive assessment tools, and personalized therapeutic strategies. The previous set of hypotheses, which focused on the complexity of mechanisms, and these hypotheses share a reliance on cutting-edge technologies, but these hypotheses differ in their emphasis on patient variability rather than on cellular heterogeneity and molecular interactions. Both sets of hypotheses aim to enhance our understanding and management of cardiotoxicity but from different angles—one at the cellular and mechanistic level and the other at the patient-specific and personalized level.

Our literature search found publications for only 3 (15%) of the 20 hypotheses, with the highest number being 15 publications for the hypothesis that conducting large-scale genome-wide association studies can identify common genetic variants that increase the risk of cardiotoxicity.

Multimedia Appendix 2 shows our evaluation results. Of the 20 hypotheses, 3 (15%) were rated as highly novel (score of 5), 15 (75%) as moderately novel (score of 4), and 2 (10%) as moderately innovative but less novel (score of 3). Our group awarded a perfect score of 5 to 3 (15%) of the 20 hypotheses, including those involving multi-omics approaches, CRISPR technology for patient-specific models, and predictive biomarkers from blood-based assays. The average score across these 20 hypotheses was 4.05 (SD 0.51), reflecting a generally high novelty level, particularly for hypotheses leveraging AI and personalized medicine approaches.

For the 20 hypotheses addressing the challenge of variability among patients (Multimedia Appendix 2), the group scores were as follows: 4, 3, 4, 4, 4, 5, 4, 4, 4, 4, 4, 3, 4, 4, 5, 5, 4, 4, 4, and 4. The total score was 81, yielding a mean score of 4.05 (SD 0.51). The median score was 4 (IQR 4.00-4.00). Preliminary analysis of interrater reliability using Fleiss κ yielded an estimated value of 0.58, indicating moderate agreement among the evaluators.

#### Lack of Detection Sensitivity

The 20 hypotheses proposed to overcome the challenge of the lack of detection sensitivity in cardiotoxicity research involve innovative applications of advanced technologies such as nanotechnology, hyperpolarized magnetic resonance imaging, liquid biopsy techniques, single-cell transcriptomics, machine learning, and wearable devices, among others (more details are provided under “Hypotheses to overcome the challenge of the lack of detection sensitivity in cardiotoxicity research” in Multimedia Appendix 1). These hypotheses are novel in their approach to enhancing the early detection of cardiotoxicity by identifying subtle and low-abundance biomarkers, leveraging high-resolution imaging, and integrating AI-driven analysis. The significance lies in their potential to revolutionize early diagnosis, allowing for timely intervention and personalized treatment adjustments, thereby improving patient outcomes. These hypotheses share similarities with those addressing the complexity of mechanisms and patient variability in their use of cutting-edge technologies and AI to deepen our understanding and improve prediction capabilities. However, they differ in focus: this set aims specifically at enhancing detection sensitivity, while the previous sets focused on understanding cellular heterogeneity and genetic variability. Together, these approaches offer a comprehensive strategy to tackle cardiotoxicity from multiple angles, ultimately advancing the field significantly.

Multimedia Appendix 3 shows the results of the evaluation of the 20 hypotheses. Of these 20 hypotheses, 4 (20%) were rated as highly novel (score of 5), 13 (65%) as moderately novel (score of 4), and 3 (15%) as slightly novel (score of 3). The average group score was 4.05 (SD 0.61), suggesting that many hypotheses were recognized for their innovative approaches to enhancing detection sensitivity in cardiotoxicity. These results indicate significant innovation in the application of advanced imaging, biosensors, and AI-driven analysis to improve early detection capabilities. The literature search result of zero publications for these hypotheses is an additional indicator of their novelty.

For the 20 hypotheses addressing the challenge of the lack of detection sensitivity (Multimedia Appendix 3), the group scores were as follows: 4, 4, 3, 4, 5, 5, 4, 3, 4, 5, 4, 4, 4, 4, 4, 4, 4, 3, 5, and 4. The total score was 81, yielding an average score of 4.05 (SD 0.61). The median score was 4 (IQR 4.00-4.00). The estimated Fleiss κ was 0.65, reflecting a good level of interrater agreement.

#### Lack of Reliable Biomarkers 

The 20 hypotheses proposed to overcome the challenge of the lack of reliable biomarkers in cardiotoxicity research involve a range of innovative approaches, including multi-omics techniques, CRISPR-based screening, scRNA-seq, noncoding RNA studies, and advanced glycomics (more details are provided under “Hypotheses to overcome the challenge of the lack of reliable biomarkers in cardiotoxicity research” in Multimedia Appendix 1). These hypotheses are novel because they use cutting-edge technologies and integrative bioinformatics to uncover new molecular markers, leveraging genetic, proteomic, metabolomic, and epigenetic data. Their significance lies in the potential to discover robust, noninvasive biomarkers that can predict and monitor cardiotoxicity with high accuracy, thereby improving early detection and patient outcomes. These hypotheses differ from those addressing the complexity of mechanisms, which focused on understanding cellular and molecular interactions; and from those targeting patient variability, which aimed at personalized risk assessment. They also differ from the hypotheses aimed at improving detection sensitivity, which concentrated on enhancing the sensitivity of existing diagnostic tools. Together, these comprehensive approaches collectively advance the field by tackling cardiotoxicity from multiple angles—mechanistic understanding, personalized prediction, enhanced detection, and reliable biomarker identification.

Multimedia Appendix 4 shows the results of the evaluation of the 20 hypotheses. Of these 20 hypotheses, 2 (10%) were rated as highly novel (score of 5), 15 (75%) as moderately novel (score of 4), 2 (20%) as moderately innovative but less novel (score of 3), and 1 (5%) as low novelty (score of 2). High group scores of 5 were given to hypotheses such as those using multi-omics approaches and CRISPR-based screening. However, some of the hypotheses (1/20, 5%), such as those using metabolomic profiling, received lower scores, resulting in an overall average group score of 3.9 (SD 0.64). This indicates that while several hypotheses were seen as highly novel, others were considered less groundbreaking in identifying reliable biomarkers.

Publications were found for 5 (25%) of the 20 hypotheses, with the number of publications ranging from 0 to 50. The hypothesis involving metabolomic profiling had the largest number of publications (50).

For the 20 hypotheses (Multimedia Appendix 4), the group scores were as follows: 4, 4, 3, 3, 4, 4, 4, 4, 4, 4, 4, 4, 4, 4, 4, 4, 4, 4, 4, and 4. The total score was 78, resulting in a mean score of 3.90 (SD 0.64). The median score was 4 (IQR 4.00-4.00). Given the clustering of scores, the estimated Fleiss κ was 0.80, indicating high interrater reliability.

#### Limitations of Animal Models

The 20 hypotheses proposed to overcome the challenge of the limitations of animal models in cardiotoxicity research focus on developing advanced human-relevant models, such as induced pluripotent stem cell–derived cardiomyocytes, 3D bioprinted heart tissues, organ-on-a-chip systems, and genetically modified or chimeric animal models (more details are provided under “Hypotheses to overcome the challenge of the limitations of animal models in cardiotoxicity research” in Multimedia Appendix 1). These approaches are novel because they leverage cutting-edge technologies such as CRISPR-Cas9, multiorgan-on-a-chip, and human cardiac organoids to create more accurate and predictive models of human cardiotoxic responses. The significance of these hypotheses lies in their potential to enhance the translational relevance of preclinical studies, reduce reliance on animal models, and improve the prediction of cardiotoxic effects in humans. These approaches could significantly impact research by providing more reliable and human-specific data, leading to better drug safety and efficacy assessments. Compared to the previous sets of hypotheses, these hypotheses focus on improving the relevance and accuracy of preclinical models rather than on understanding mechanisms, personalizing risk prediction, enhancing detection sensitivity, or discovering biomarkers. However, they share a common goal of advancing cardiotoxicity research through innovative and technologically advanced methods. Together, these comprehensive approaches address various facets of cardiotoxicity, ultimately aiming to improve patient safety and treatment outcomes.

Our group evaluated 20 hypotheses aimed at overcoming the challenge of the limitations of animal models in cardiotoxicity research (Multimedia Appendix 5). Of these 20 hypotheses, 3 (15%) were rated as highly novel (score of 5), 14 (70%) as moderately novel (score of 4), 2 (10%) as moderately innovative but less novel (score of 3), and 1 (5%) as low novelty (score of 2).

Publications were found for 7 (35%) of the 20 hypotheses, with the number of publications ranging from 0 to 35. The highest number of publications (35) were found for the hypothesis on computational modeling and simulations, suggesting that this area is relatively well-established and that the hypothesis may therefore be considered to have lower novelty.

For the 20 hypotheses addressing the challenge of the limitations of animal models (Multimedia Appendix 5), the group scores were as follows: 5, 5, 4, 4, 4, 3, 3, 2, 4, 4, 4, 4, 4, 4, 4, 4, 4, 5, 5, and 4. The total score was 80, yielding a mean score of 3.95 (SD 0.69). The median score was 4 (IQR 4.00-4.00). The estimated Fleiss κ for these evaluations was 0.70, suggesting moderate to high agreement among the evaluators.

### Ability of ChatGPT With GPT-4o to Develop Experimental Plans to Test Selected Hypotheses

#### Overview

The 5 selected hypotheses from each set—addressing the complexity of mechanisms, variability among patients, the lack of detection sensitivity, the lack of reliable biomarkers, and the limitations of animal models—represent innovative approaches to overcoming significant challenges in cardiotoxicity research (the individual sets of hypotheses are presented in Multimedia Appendix 1). These 5 hypotheses share a common goal of enhancing the accuracy and predictive power of cardiotoxicity assessments through advanced technologies and comprehensive data analysis. However, they differ in their specific methodologies: using scRNA-seq to understand cellular heterogeneity, integrating genetic profiles with AI for personalized risk prediction, applying machine learning to ECG data to improve detection sensitivity, using multi-omics approaches for biomarker discovery, and developing 3D bioprinted heart tissues for human-relevant models.

Our group’s evaluation of the 30 dimensions of the experimental plans for these 5 hypotheses provided by ChatGPT with GPT-4o revealed consistent strengths in the background, rationale, and alternative approaches, with most of the hypotheses (4/5, 80% to 5/5, 100%) receiving scores of ≥4 in these areas (Table 3). However, the experimental design was generally rated lower, with most of the hypotheses (4/5, 80%) scoring 2 or 3, indicating room for improvement in the design aspects. Expected outcomes and potential pitfalls received mixed reviews, with scores typically being 3 or 4. Overall, while the hypotheses were well supported by the background and rationale, the experimental design emerged as the weakest aspect across all 5 hypotheses, suggesting a need for refinement in this area to strengthen future experiments involving the training of ChatGPT with GPT-4o.

**Figure 2:**
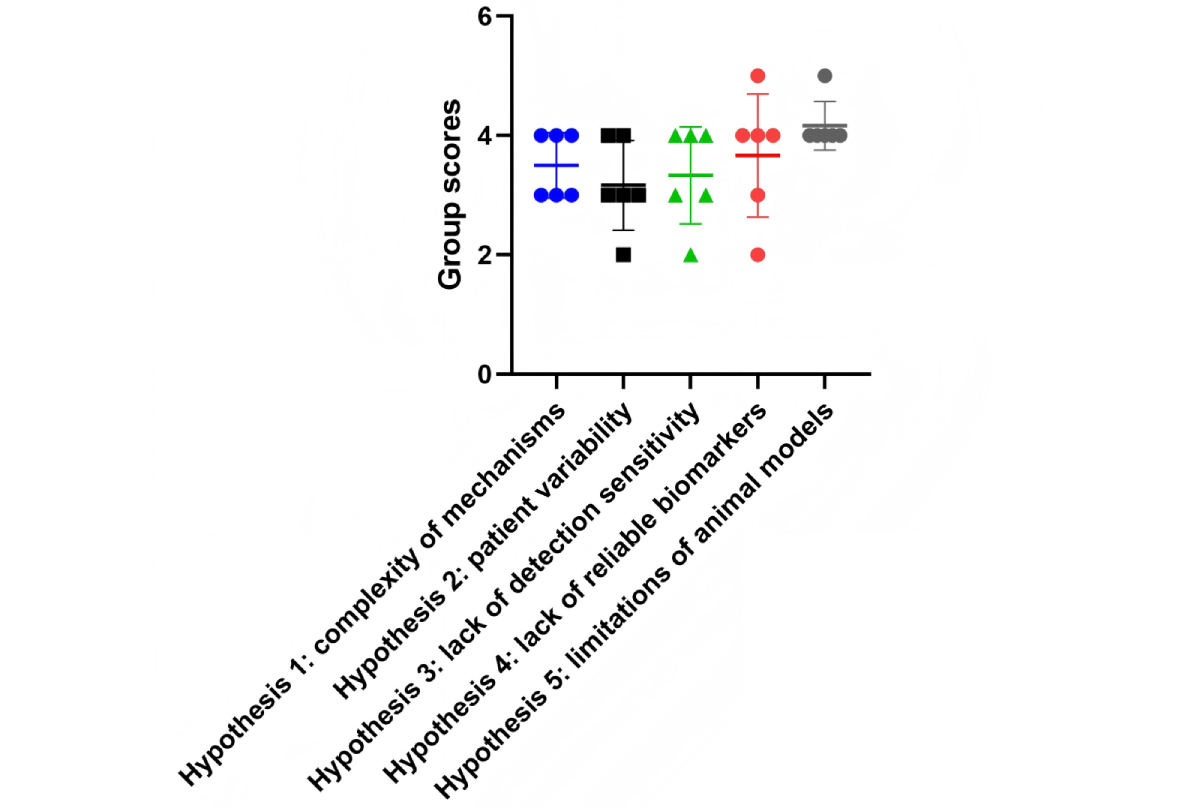
Distribution of group scores for research plans generated by ChatGPT with GPT-4o across 5 hypotheses for challenges in cardiotoxicity research across multiple assessment dimensions.

Table 3 and Figure 2 summarize the evaluation of the research plans generated by ChatGPT with GPT-4o across 5 challenge domains, each assessed on 6 dimensions (background, rationale, experimental design, expected outcomes, potential pitfalls, and alternative approaches). For the “complexity of mechanisms” domain, the scores were 4, 3, 3, 3, 4, and 4, yielding an average of 3.50 (SD 0.55; median 3.5, IQR 3.00-4.00). For the “patient variability” domain, the scores (3, 3, 2, 3, 4, and 4) resulted in an average of 3.17 (SD 0.75; median 3, IQR 2.75-4.00). The “detection sensitivity” domain had scores of 4, 4, 2, 4, 3, and 3, with an average of 3.33 (SD 0.82; median 3.5, IQR 2.75-4.00). For the “reliable biomarkers” domain, the scores (4, 4, 2, 3, 4, and 5) produced an average of 3.67 (SD 1.03; median 4, IQR 2.75-4.25). Finally, the “limitations of animal models” domain received scores of 4, 4, 4, 4, 4, and 5, corresponding to an average of 4.17 (SD 0.41; median 4, IQR 4.00-4.25).

**Table 3:** Evaluation of the research plans generated by ChatGPT with GPT-4o for each hypothesis.

Hypotheses and dimensions		Evaluator 1^a^ score	Evaluator 2^b^ score	Evaluator 3^c^ score	Group consensus score
**1. Overcome the challenge of the complexity of mechanisms**					
	Background	4	4	3	4
	Rationale	5	5	3	3
	Experimental design	5	4	3	3
	Expected outcomes	4	4	3	3
	Potential pitfalls	5	5	4	4
	Alternative approaches	4	4	4	4
**2. Overcome the challenge of variability among patients**					
	Background	4	4	3	3
	Rationale	4	4	3	3
	Experimental design	4	4	2	2
	Expected outcomes	4	4	3	3
	Potential pitfalls	4	4	4	4
	Alternative approaches	4	4	4	4
**3. Overcome the challenge of the lack of detection sensitivity**					
	Background	5	5	4	4
	Rationale	5	4	3	4
	Experimental design	4	3	2	2
	Expected outcomes	4	4	4	4
	Potential pitfalls	4	4	3	3
	Alternative approaches	4	4	3	3
**4. Overcome the challenge of the lack of reliable biomarkers**					
	Background	5	5	4	4
	Rationale	5	5	4	4
	Experimental design	5	3	2	2
	Expected outcomes	4	4	3	3
	Potential pitfalls	5	5	4	4
	Alternative approaches	5	5	4	5
**5. Overcome the challenge of the limitations of animal models**					
	Background	4	4	4	4
	Rationale	5	5	4	4
	Experimental design	5	4	4	4
	Expected outcomes	4	4	4	4
	Potential pitfalls	5	5	4	4
	Alternative approaches	5	5	4	5

^a^Author YL (MD and PhD, professor).

^b^Author TG (MD, final-year PhD candidate).

^c^Author CY (MD, first-year PhD student).

#### Complexity of Mechanisms

ChatGPT with GPT-4o selected the hypothesis that scRNA-seq can dissect heterogeneous cellular responses to cardiotoxic agents because of its novel approach in leveraging cutting-edge technology to address a critical gap in cardiotoxicity research. This hypothesis stands out due to its innovative use of scRNA-seq to reveal distinct subpopulations of cardiomyocytes, offering insights that traditional bulk RNA sequencing cannot provide. Its feasibility is supported by the increasing accessibility and reliability of scRNA-seq and the availability of advanced bioinformatics tools for data analysis. If successful, this research could have a profound impact by identifying specific molecular signatures and pathways associated with cardiotoxicity, leading to the development of targeted therapies and biomarkers for early detection. This would significantly enhance drug safety and efficacy, ultimately improving patient outcomes in clinical settings.

Our group’s evaluation resulted in scores of 4 for the background, 3 for the rationale, 3 for the experimental design, 3 for the expected outcomes, 4 for the potential pitfalls, and 4 for the alternative approaches.

We assigned a score of 4 to the description of the background. ChatGPT with GPT-4o provided a clear explanation of the relevance of cardiotoxicity as a major side effect and the potential of scRNA-seq to offer unprecedented insights into cellular heterogeneity, although there was room for slight improvement in depth or detail. The rationale was considered solid but not particularly innovative. While the need to go beyond bulk RNA sequencing is well justified, the proposed use of scRNA-seq was somewhat standard, without a strong, novel angle or a particularly compelling argument, leading us to give it a score of 3. The experimental design was sound but raised some concerns. The design is straightforward and feasible, but the challenges related to scRNA-seq, such as technical difficulties or the complexity of data analysis, were not fully addressed, which led us to assign a slightly lower score. The expected outcomes were logical but not particularly groundbreaking. We felt that because the identification of subpopulations and molecular signatures is expected, the outcomes might not offer surprising or highly impactful new insights, leading to a moderate score. We rated the potential pitfalls highly because ChatGPT with GPT-4o thoroughly acknowledges and addresses the potential challenges associated with the experimental approach. Similarly, the alternative approaches were rated well due to their thoroughness and feasibility. Our group appreciated the proactive thinking in suggesting alternative methods and validations to ensure the robustness and translational relevance of the findings. However, a perfect score was withheld when the alternatives were somewhat standard or when additional novel approaches could have been proposed.

Overall, we recognized the solid foundation and well-considered plan of the hypothesis, balanced with some reservations about the innovation and complexity of the proposed work.

#### Variability Among Patients

ChatGPT with GPT-4o selected the hypothesis of integrating patient-specific genetic profiles with AI algorithms to predict individual susceptibility to cardiotoxicity due to its innovative approach in harnessing advanced genomics and machine learning to address patient variability in drug responses. The novelty of this hypothesis lies in its potential to move beyond one-size-fits-all treatment strategies by leveraging AI to analyze complex genetic data, thereby tailoring cardiotoxicity risk assessments and treatment plans to individual patients. Its feasibility is supported by the increasing availability and affordability of whole-genome sequencing and the advancements in AI algorithms capable of handling large datasets. If successful, this approach could revolutionize personalized medicine, significantly reducing adverse drug reactions and improving patient outcomes by enabling clinicians to make more informed decisions based on genetic risk profiles.

This hypothesis is similar to the previously selected hypothesis to overcome the challenge of the complexity of mechanisms in that both leverage cutting-edge technologies to provide deeper insights into cardiotoxicity. While the previous hypothesis focused on understanding cellular heterogeneity and molecular interactions using scRNA-seq, this hypothesis emphasizes the individual variability among patients by integrating genetic data with AI. Both aim to enhance predictive accuracy and personalized care, but they tackle the issue from different angles—one at the cellular and mechanistic level and the other at the patient-specific and genetic level. Together, these approaches represent a comprehensive strategy to understand and mitigate cardiotoxicity in a more precise and personalized manner.

We graded the second hypothesis as follows: 3 for the background, 3 for the rationale, 2 for the experimental design, 3 for the expected outcomes, 4 for the potential pitfalls, and 4 for the alternative approaches.

The background was solid but not particularly novel. The importance of cardiotoxicity and the potential of genomics and AI are well-established topics; hence, we found the background sufficient but not particularly compelling or groundbreaking, which led to the moderate score. The rationale received a similar score, likely because while the integration of AI with genetic data is a logical next step, it did not present a highly innovative or original idea.

The low score of 2 for the experimental design reflects the significant concerns of our group, which included the following:

1. The design proposes using AI to analyze complex genetic data, which is challenging and was not sufficiently detailed or realistic in the plan. We felt that the plan lacked clarity on how the AI algorithms would be developed, validated, and implemented in a clinical setting, particularly given the inherent complexities of genetic data.
2. We also had concerns about the feasibility of enrolling a sufficient and diverse cohort, obtaining comprehensive genetic and clinical data, and developing a reliable AI model within the scope of the study. The design did not provide enough detail on how these challenges would be practically addressed.
3. The design’s approach to validation seemed insufficient, especially regarding the generalizability of the model across diverse populations. We felt that this was a critical weakness.

The expected outcomes received a score of 3, likely because they were seen as reasonable but not particularly innovative or surprising. While predicting cardiotoxicity and improving patient outcomes are valuable goals, the group might have felt that these outcomes are somewhat standard expectations for a study of this nature, leading to a moderate score.

The potential pitfalls section was rated higher because it thoughtfully acknowledges the challenges associated with genetic data complexity, data privacy, model generalizability, and integration into clinical practice. Similar to the potential pitfalls, the alternative approaches were rated well because they were comprehensive and well considered.

Overall, our assessment was that while the hypothesis is grounded in a strong concept and well-considered alternative approaches, the experimental design raised significant concerns about feasibility, clarity, and the practical implementation of the AI model, leading to the notably lower score in this area.

#### Lack of Detection Sensitivity

ChatGPT with GPT-4o selected the hypothesis of developing machine learning algorithms to analyze ECG data for detecting subtle and early changes in cardiac electrical activity associated with cardiotoxicity due to its innovative approach and high feasibility. This hypothesis leverages the widespread use of ECGs in clinical practice and the advanced capabilities of machine learning to enhance detection sensitivity. Its novelty lies in applying sophisticated AI techniques to a traditionally well-established diagnostic tool, potentially uncovering early cardiotoxic signs that conventional analysis might miss. The feasibility is supported by the growing accessibility of machine learning frameworks and the availability of large ECG datasets. If successful, this approach could revolutionize cardiotoxicity monitoring by enabling earlier detection and intervention, thereby improving patient outcomes and reducing long-term cardiac damage.

This hypothesis shares similarities with the previously selected hypotheses addressing the complexity of mechanisms and variability among patients in its use of advanced technologies such as AI and machine learning. However, it differs in its specific focus on enhancing detection sensitivity rather than on understanding cellular mechanisms or individual genetic variability. While the hypothesis on scRNA-seq aims to dissect cellular heterogeneity and the one on integrating genetic profiles with AI targets personalized susceptibility, this hypothesis focuses on improving the sensitivity of an existing diagnostic tool to detect early cardiac changes. Together, these hypotheses offer a comprehensive approach to tackling cardiotoxicity from different angles: mechanistic understanding, personalized prediction, and enhanced early detection.

This hypothesis was graded as follows: 4 for the background, 4 for the rationale, 2 for the experimental design, 4 for the expected outcomes, 3 for the potential pitfalls, and 3 for the alternative approaches.

Similar to the grading of the previous 2 hypotheses, the background was rated highly because it effectively highlights the importance of identifying reliable biomarkers for cardiotoxicity and underscores the limitations of current methods. The rationale also received a strong score due to the convincing argument that integrating multi-omics data can provide a more comprehensive understanding of the molecular changes associated with cardiotoxicity. The experimental design again received a low score, reflecting the significant concerns of our team, which included the following:

1. The design involves integrating data from genomics, proteomics, and metabolomics, which is highly complex and overly ambitious. We have concerns about the feasibility of managing, integrating, and analyzing such large and diverse datasets effectively within the scope of the study.
2. We had concerns about the practicality and consistency of collecting high-quality samples across different omics platforms. We felt that the plan lacked sufficient detail on how sample variability would be controlled or how high standards would be maintained throughout the study.
3. We found the approach to data integration and validation underdeveloped or lacking in detail. The process of correlating multi-omics data with clinical outcomes and validating biomarkers in independent cohorts seemed insufficiently robust or not well defined, leading to doubts about the reliability and generalizability of the findings.

The expected outcomes were rated positively because they align well with the study’s objectives and promise significant advancements in biomarker discovery. The potential pitfalls section received a moderate score because while the challenges associated with data complexity, sample variability, and validation were acknowledged, the group’s confidence in the proposed solutions was lukewarm. Similar to the potential pitfalls, the alternative approaches were seen as reasonable but not particularly innovative or detailed.

Overall, our assessment reflects a recognition of the strong conceptual foundation and potential impact of the hypothesis but with significant concerns about the practical execution of the experimental design, particularly in managing the complexity of multi-omics data and ensuring the reliability and validity of the findings.

#### Lack of Reliable Biomarkers

ChatGPT with GPT-4o selected the hypothesis of using multi-omics approaches (ie, genomics, proteomics, and metabolomics) to identify novel and reliable biomarkers for cardiotoxicity due to its innovative potential to provide a comprehensive understanding of the molecular alterations associated with cardiotoxicity. The novelty of this hypothesis lies in its integrative approach, combining data from different omics layers to capture a holistic view of the biological changes induced by cardiotoxic agents. This multidimensional perspective is more likely to identify sensitive and specific biomarkers than traditional single-omics methods. The feasibility of this approach is supported by advances in high-throughput technologies and bioinformatics tools, making it practical to generate and analyze large-scale multi-omics datasets. If successful, this research could revolutionize the detection and monitoring of cardiotoxicity, leading to earlier interventions and personalized treatment strategies, ultimately improving patient outcomes.

Compared to the previously selected hypotheses, this approach shares similarities in leveraging advanced technologies and comprehensive data analysis to address cardiotoxicity. However, it differs in its specific focus on biomarker discovery through multi-omics integration. The hypothesis addressing the complexity of mechanisms used scRNA-seq to understand cellular heterogeneity, while this multi-omics approach targets biomarker identification across different biological layers. The hypothesis on patient variability focused on integrating genetic profiles with AI for personalized risk prediction, whereas this hypothesis aims to discover universal biomarkers applicable across patient populations. Finally, the hypothesis on enhancing detection sensitivity with machine learning and ECG data centered on improving existing diagnostic tools, while this hypothesis seeks to establish new biomarkers altogether. Collectively, these approaches provide a multifaceted strategy to tackle cardiotoxicity from different angles, enhancing our ability to predict, detect, and manage this adverse effect.

We graded the dimensions of the experimental plan for this hypothesis as follows: 4 for the background, 4 for the rationale, 2 for the experimental design, 3 for the expected outcomes, 4 for the potential pitfalls, and 5 for the alternative approaches.

Both the background and rationale were given a score of 4 because ChatGPT with GPT-4o effectively underscores the significance of cardiotoxicity and the potential of multi-omics approaches to improve biomarker discovery, while presenting convincing arguments that integrating genomics, proteomics, and metabolomics can provide a more comprehensive understanding of cardiotoxicity.

Again, a low score of 2 was given to the experimental design mainly for the following reasons:

1. The design proposes a complex and ambitious plan to integrate multi-omics data, which raised concerns about the feasibility of executing such a broad approach within the constraints of time, resources, and technical capability. We felt that the experimental design was too complex and lacked practical details on how the integration and analysis would be managed effectively.
2. We had concerns about the practicality and consistency of collecting and processing samples across different omics platforms. We felt that the plan did not adequately address how sample quality and variability would be controlled, which is critical for the reliability of multi-omics data.
3. We found the proposed validation process for the biomarkers insufficiently detailed and lacking in rigor.

The expected outcomes were rated moderately (score of 3) because while they are logical and align with the study’s objectives, the group perceived them as somewhat standard or predictable. The outcomes, such as identifying novel biomarkers and improving patient outcomes, are valuable but were not seen as particularly groundbreaking.

The potential pitfalls section received a high score because of the detailed consideration of major potential issues and the proposed solutions, which reflect a realistic understanding of the study’s challenges.

Our group found the alternative approaches to the plan to be exceptionally well considered and robust. The alternative approaches were seen as comprehensive and practical, offering clear and feasible strategies to address potential challenges.

In summary, while we recognized the strong conceptual foundation and thorough consideration of potential challenges in this hypothesis, the experimental design’s complexity and feasibility issues led to a lower score. However, the well-developed alternative approaches significantly bolstered our confidence in the study’s potential to overcome these challenges, earning a top score in this category.

#### Limitations of Animal Models

ChatGPT with GPT-4o selected the hypothesis of using 3D bioprinted human heart tissues for cardiotoxicity testing due to its innovative approach and significant potential to address the limitations of current preclinical models. The novelty of this hypothesis lies in its ability to replicate the structural and functional characteristics of human hearts, providing a more accurate and physiologically relevant model for cardiotoxicity studies. The feasibility of this approach is supported by recent advancements in 3D bioprinting technology and stem cell research, which enable the creation of complex, multicellular heart tissues. If successful, this model could revolutionize cardiotoxicity testing by offering a more reliable and predictive alternative to traditional animal models, leading to safer and more effective therapeutic agents.

The previously selected hypotheses and this hypothesis share similarities in leveraging cutting-edge technologies and aiming to improve the predictive accuracy of cardiotoxicity assessments. However, this hypothesis differs in its focus on creating a tangible, 3D human heart tissue model as opposed to understanding cellular mechanisms (scRNA-seq), personalizing risk prediction (AI with genetic profiles), enhancing detection sensitivity (machine learning on ECG data), or discovering biomarkers (multi-omics approaches).

This hypothesis was graded as follows: 4 for the background, 4 for the rationale, 4 for the experimental design, 4 for the expected outcomes, 4 for the potential pitfalls, and 5 for the alternative approaches. This is the highest rated of the 5 hypotheses. In the following paragraphs, we provide an explanation for the evaluation, particularly the score of 4 for the experimental design and the score of 5 for the alternative approaches.

The background was rated highly because it effectively highlights the limitations of traditional animal models and the potential of 3D bioprinting technology to address these challenges.

The rationale also received a high score because it convincingly argues for the superiority of 3D bioprinted tissues over traditional 2D cultures and animal models. We found the rationale compelling because it emphasizes the physiological relevance of, and potential for, improved predictive accuracy in cardiotoxicity testing. The experimental design was rated well, indicating that the proposed methods were sound and feasible. The design’s clarity, organization, and attention to detail contributed to this positive evaluation. The expected outcomes were rated highly because they align well with the hypothesis and promise significant advancements in cardiotoxicity testing. The score of 4 reflects strong confidence in the expected outcomes, although some aspects, such as the validation process, could be further refined. The potential pitfalls section received a high score because it thoughtfully addresses the key challenges associated with 3D bioprinting, such as cell viability, reproducibility, scalability, and validation. The perfect score for the alternative approaches suggests that this aspect of the plan is exceptionally well considered and robust. We felt that ChatGPT with GPT-4o, which had been instructed to act as a biomedical research scientist, had thoroughly thought through the possible obstacles and proposed effective, realistic solutions, significantly enhancing the overall viability of the study.

In summary, our group strongly endorsed this hypothesis and experimental plan. The slightly lower score for the experimental design suggests minor areas for improvement, but overall, this plan was considered the best among the 5 plans. A detailed interpretation and comparison of hypothesis quality across domains are presented in the Discussion section.

## Discussion

### Principal Findings

Our study involved using ChatGPT with GPT-4o to generate hypotheses addressing 5 major challenges in cardiotoxicity research: the complexity of mechanisms, variability among patients, the lack of detection sensitivity, the lack of reliable biomarkers, and the limitations of animal models. ChatGPT with GPT-4o was first tasked with producing multiple hypotheses for each challenge [30-34]. These hypotheses were then independently evaluated for novelty and feasibility by 3 experts. After the evaluation, ChatGPT with GPT-4o selected the best hypothesis from each category and provided a detailed experimental plan, including background, rationale, experimental design, expected outcomes, potential pitfalls, and alternative approaches. Our study’s overall goal was to assess the ability of ChatGPT with GPT-4o to generate innovative and impactful research hypotheses that could advance the field of cardiotoxicity.

The most significant finding of our study is the demonstration of the ability of ChatGPT with GPT-4o to generate a large number of hypotheses, with a notable portion of them (13/96, 14%) being highly novel and innovative. Of the 96 generated hypotheses, 13 (14%) were rated as highly novel (score of 5) and 62 (65%) as moderately novel (score of 4), highlighting the tool’s potential in contributing fresh ideas to cardiotoxicity research. The high level of innovation, particularly in leveraging advanced technologies such as AI, multi-omics, CRISPR, and 3D bioprinting, suggests that AI-assisted hypothesis generation could significantly advance research in this field [35-37].

### Comparison of Hypotheses and Research Impact

Among the 5 selected hypotheses, the one addressing the limitations of animal models—through the use of 3D bioprinted human heart tissues—was rated the highest across all evaluation criteria. This reflects the growing recognition of advanced human-relevant models as a transformative solution for improving preclinical cardiotoxicity testing. The physiological relevance of these models provides a direct alternative to traditional animal systems, potentially enhancing the predictive accuracy of drug safety assessments. By contrast, hypotheses that focused on patient variability and detection sensitivity, although conceptually innovative, were rated lower in feasibility—particularly in terms of experimental design and implementation. These discrepancies feature a critical consideration: while AI-generated hypotheses can be highly creative, their practical applicability still depends on realistic methodological planning and execution. Overall, these findings suggest that an optimal path forward may lie in combining AI’s strength in ideation with human expertise in refining, validating, and operationalizing these ideas. The 5 selected hypotheses collectively represent a comprehensive strategy to tackle cardiotoxicity from multiple dimensions, including mechanistic insight, personalized prediction, biomarker discovery, early detection, and model refinement, offering promising avenues to improve both the safety and efficacy of therapeutics.

The impact of this research could be substantial because it not only introduces new avenues for exploration in cardiotoxicity but also suggests that AI could play a crucial role in overcoming some of the most challenging aspects of drug safety assessment. This study has the potential to significantly impact the 5 major challenges in cardiotoxicity research by offering innovative solutions tailored to each challenge. For the complexity of mechanisms, it introduces advanced approaches such as scRNA-seq to dissect cellular responses [37]. In addressing variability among patients, it emphasizes the integration of AI with genetic profiling for personalized risk prediction. To overcome detection sensitivity issues, the study suggests leveraging machine learning with ECG data to enhance early detection capabilities. For the challenge of identifying reliable biomarkers, it proposes multi-omics integration to discover more precise and comprehensive biomarkers. Finally, to address the limitations of animal models, the study advocates for the use of 3D bioprinted heart tissues, offering a more accurate human-relevant platform for cardiotoxicity testing. Collectively, these innovative approaches could advance the understanding, prediction, and management of cardiotoxicity, ultimately improving patient outcomes.

One key concern is the potential for AI to be used as a shortcut for scientific inquiry, potentially leading to a decline in critical thinking and originality, particularly among early-career researchers or students. Overreliance on AI-generated hypotheses without appropriate validation could result in poorly conceptualized studies and a dilution of scientific rigor. In addition, AI-generated content may inadvertently introduce factual inaccuracies or misinterpretations of existing knowledge, further emphasizing the need for human oversight [38,39].

Another ethical issue involves scientific attribution and plagiarism [26]. AI models such as ChatGPT with GPT-4o generate text based on preexisting knowledge and training data, but they do not independently create novel scientific ideas in the way human researchers do. Therefore, proper disclosure of AI involvement, as demonstrated in this study, is essential to maintain transparency in scientific authorship. Recent discussions in cellular and molecular bioengineering have highlighted the importance of establishing clear ethical guidelines for AI-generated research content, particularly in biomedical sciences. We support the ongoing development of such guidelines to ensure that AI is used responsibly as a research aid rather than as a substitute for human expertise.

Although using an internet-enabled tool powered by GPT-4, such as the Microsoft Bing chatbot, could allow for an automated literature search to evaluate the novelty of AI-generated hypotheses, in our study, we opted for human-led literature searches to ensure accuracy, context-aware evaluation, and the critical assessment of the relevance and quality of references. While AI-assisted searches may be useful, they also come with limitations, such as potential biases in search results, lack of access to paywalled journal articles, and difficulties in accurately interpreting complex biomedical literature.

In addition, AI’s ability to rapidly generate a high volume of hypotheses and research ideas raises concerns about overwhelming funding agencies such as the National Institutes of Health and the National Science Foundation with AI-generated proposals, potentially leading to unintended consequences in grant evaluation and scientific funding distribution. While AI can enhance idea generation, the human-led process of refining, evaluating, and prioritizing research directions remains indispensable.

### Future Directions

In light of these concerns, we emphasize that AI should be viewed as an assistive tool that enhances human creativity rather than replaces it. All hypotheses generated in this study were subjected to rigorous expert evaluation, ensuring that only scientifically sound and feasible ideas were considered. Future work should explore best practices for integrating AI into scientific research while maintaining academic integrity and critical thinking as core principles of hypothesis-driven inquiry. Testing the hypotheses produced by ChatGPT with GPT-4o in real clinical sites or laboratories is essential for evaluating the feasibility and true value of AI assistance in biomedical research. In addition, future studies may incorporate multiple AI models (eg, large-scale language models specialized in biomedical research) to compare and contrast AI-generated evaluations with human judgments.

### Limitations

While our study highlights the strengths of ChatGPT with GPT-4o in hypothesis generation, our study has several limitations. First, the output of ChatGPT with GPT-4o is limited by the data it has been trained on, potentially missing emerging trends or novel insights not yet well documented. Second, many of the experimental designs proposed by ChatGPT with GPT-4o were considered overly ambitious or lacking in practical details, raising concerns about their feasibility. Third, this study did not test the generated hypotheses in a real-world setting; therefore, their practical applicability and effectiveness remain uncertain. Fourth, while expert evaluation allowed for a nuanced and context-aware assessment of scientific novelty, we acknowledge that the inclusion of more objective and standardized evaluation frameworks—such as automated keyword matching, citation overlap analysis, or algorithmic scoring—may improve the reproducibility and generalizability of future studies. A more objective and standardized approach could enhance the reproducibility of our findings. Fifth, the scoring distribution showed a clustering around the score of 4, which may reflect the limited discriminative resolution of the 5-point scale. In future work, we aim to refine the scoring rubric and consider extended or weighted evaluation frameworks to improve scoring granularity. Finally, while prompt engineering strategies were used to reduce inconsistency across queries, occasional prompt drift was observed. This reflects a known limitation of current large language models and highlights the importance of standardized, repeatable prompts for real-world deployment in health care settings.

### Conclusions

This study presents preliminary evidence that large language models such as ChatGPT with GPT-4o may serve as useful tools for generating innovative research hypotheses in cardiotoxicity. By evaluating the novelty and feasibility of AI-generated ideas, we demonstrated the potential value of AI-assisted ideation in the early stages of biomedical research. However, these findings are based on simulated outputs and expert review and should not be interpreted as a validation of real-world scientific outcomes. Further studies involving empirical testing and clinical application are needed to assess the practical impact of such AI-generated hypotheses. Our work underscores the importance of integrating human expertise with AI tools to promote responsible and rigorous scientific discovery.

## Data Availability

All data generated or analyzed during this study are included in this published paper and its supplementary information files.

## Appendix

Multimedia Appendix 1 Evaluation criteria and hypotheses generated by ChatGPT with GPT-4o to overcome 5 major challenges in cardiotoxicity research.

Multimedia Appendix 2 Evaluation of hypotheses to overcome the challenge of variability among patients in cardiotoxicity research.

Multimedia Appendix 3 Evaluation of hypotheses to overcome the challenge of the lack of detection sensitivity in cardiotoxicity research.

Multimedia Appendix 4 Evaluation of hypotheses to overcome the challenge of the lack of reliable biomarkers in cardiotoxicity research.

Multimedia Appendix 5 Evaluation of hypotheses to overcome the challenge of the limitations of animal models in cardiotoxicity research.

## Abbreviations

AI: artificial intelligence
CRISPR: clustered regularly interspaced short palindromic repeats
ECG: electrocardiogram
scRNA-seq: single-cell RNA sequencing
