# Supplementary material for: AI-Assisted Hypothesis Generation to Address Challenges in Cardiotoxicity Research: Simulation Study Using ChatGPT With GPT-4o
Source: J Med Internet Res. 2025 May 15;27:e66161. doi: 10.2196/66161 (PMC12123237; doi:10.2196/66161)
Supplement: Multimedia Appendix 2 [file jmir_v27i1e66161_app2.pdf]

Multimedia Appendix 2. Evaluation of hypotheses to overcome the challenge of variability among patients in cardiotoxicity research.

Hypotheses	Novelty	Keywords	Publications (n=18), n (%)	Evaluator 1[a] score	Evaluator 2[b] score	Evaluator 3[c] score	Group consensus score
1. Integrating patient-specific genetic profiles with AI[d] algorithms can predict individual susceptibility to cardiotoxicity, enabling personalized treatment plans	High	"genetic profiles," "AI," "cardiotoxicity," "personalized treatment"	0 (0)	4	4	4	4
2. Using patient-derived cardiomyocytes from diverse genetic backgrounds in high-throughput screening can identify genetic variants associated with increased cardiotoxicity risk	High	"patient-derived cardiomyocytes," "genetic backgrounds," "high-throughput screening," "cardiotoxicity risk"	0 (0)	3	3	4	3

3. Longitudinal studies using wearable health monitors can capture real-time cardiac data, helping to identify patient-specific patterns and early signs of cardiotoxicity	Moderate	“wearable health monitors,” “real-time cardiac data,” “cardiotoxicity patterns”	0 (0)	4	4	4	4
4. Epigenetic profiling of patients before and after drug administration can reveal markers that predict susceptibility to cardiotoxic effects	High	“epigenetic profiling,” “drug administration,” “cardiotoxicity markers”	0 (0)	4	4	4	4
5. Applying machine learning to electronic health records can uncover hidden correlations between patient demographics, comorbidities, and cardiotoxicity risk	Moderate	“machine learning,” “electronic health records, EHRs,” “patient demographics,” “comorbidities,” “cardiotoxicity risk”	0 (0)	4	5	3	4
6. Developing a multi-omics approach that combines genomics, proteomics, and	High	“multi-omics,” “genomics,”	0 (0)	5	5	4	5

metabolomics can provide a comprehensive understanding of individual variability in cardiotoxic responses		"proteomics," "metabolomics," "cardiotoxicity variability"					
7. Using CRISPR[e] technology to create patient-specific iPSC[f]-derived cardiomyocytes can help study the impact of individual genetic differences on cardiotoxicity	High	"CRISPR," "iPSC-derived cardiomyocytes," "genetic differences," "cardiotoxicity"	2 (11)	4	4	3	4
8. Investigating the role of microbiome diversity in cardiotoxicity can reveal how gut microbiota influence individual susceptibility to cardiac damage from drugs	High	"microbiome diversity," "cardiotoxicity," "gut microbiota," "cardiac damage"	1 (6)	4	4	4	4
9. Pharmacogenomics studies can identify specific gene-drug interactions that contribute to variability in cardiotoxic responses among patients	Modera te	"pharmacogenomics," "gene-drug interactions," "cardiotoxicity	0 (0)	4	4	3	4

		variability”					
10. Utilizing advanced imaging techniques, such as cardiac MRI[g], can non-invasively assess patient-specific cardiac changes and predict cardiotoxicity risk	Moderate	“advanced imaging,” “cardiac MRI,” “patient-specific cardiac changes,” “cardiotoxicity risk”	0 (0)	4	4	3	4
11. Exploring the impact of hormonal differences, such as variations in sex hormones, on cardiotoxicity can help understand gender-specific risks	Moderate	“hormonal differences,” “sex hormones,” “cardiotoxicity,” “gender-specific risks”	0 (0)	4	4	3	4
12. Conducting large-scale genome-wide association studies can identify common genetic variants that increase the risk of cardiotoxicity	Moderate	“genome-wide association studies, GWAS,” “genetic variants,” “cardiotoxicity risk”	15 (83)	5	3	3	3

13. Studying the interaction between environmental factors, such as diet and lifestyle, and genetic predisposition can provide insights into patient-specific cardiotoxicity risk	Modera te	"environmental factors," "diet," "lifestyle," "genetic predisposition," "cardiotoxicity"	0 (0)	4	4	4	4
14. Investigating the role of immune system variability in cardiotoxicity can reveal how individual differences in immune response contribute to cardiac damage	Modera te	"immune system variability," "cardiotoxicity," "immune response," "cardiac damage"	0 (0)	5	5	3	4
15. Applying personalized medicine approaches to adjust drug dosages based on individual metabolic profiles can reduce the risk of cardiotoxicity	Modera te	"personalized medicine," "drug dosages," "metabolic profiles," "cardiotoxicity"	0 (0)	5	5	4	5

16. Developing predictive biomarkers from blood-based assays can provide non-invasive tools for assessing individual cardiotoxicity risk before drug administration	High	"predictive biomarkers," "blood-based assays," "cardiotoxicity risk," "non-invasive tools"	0 (0)	5	5	3	5
17. Analyzing patient-specific variations in drug metabolism enzymes can help predict and mitigate cardiotoxicity risk	Modera te	"drug metabolism enzymes," "patient-specific variations," "cardiotoxicity risk"	0 (0)	4	4	4	4
18. Using virtual clinical trials with simulated patient populations can model and predict variability in cardiotoxic responses across diverse demographics	High	"virtual clinical trials," "simulated patient populations," "cardiotoxicity variability"	0 (0)	4	4	3	4

19. Investigating the impact of epitranscriptomic modifications, such as RNA methylation, on cardiotoxicity can uncover new layers of individual variability in drug response	High	“epitranscriptomic modifications,” “RNA methylation,” “cardiotoxicity,” “drug response”	0 (0)	4	4	4	4
20. Implementing AI-driven predictive analytics on patient genomic data can enhance the identification of at-risk individuals and tailor cardioprotective strategies accordingly	High	“AI-driven predictive analytics,” “genomic data,” “cardiotoxicity,” “cardioprotective strategies”	0 (0)	4	4	4	4

[a]Author YL (MD and PhD, professor).

[b]Author TG (MD, final-year PhD candidate).

[c]Author CY (MD, first-year PhD student).

[d]AI: artificial intelligence.

[e]CRiSPR: clustered regularly interspaced short palindromic repeats.

[f]iPSC: induced pluripotent stem cell.

[g]MRI: magnetic resonance imaging.
