# Supplementary material for: AI-Assisted Hypothesis Generation to Address Challenges in Cardiotoxicity Research: Simulation Study Using ChatGPT With GPT-4o
Source: J Med Internet Res. 2025 May 15;27:e66161. doi: 10.2196/66161 (PMC12123237; doi:10.2196/66161)
Supplement: Multimedia Appendix 3 [file jmir_v27i1e66161_app3.pdf]

Multimedia Appendix 3. Evaluation of hypotheses to overcome the challenge of the lack of detection sensitivity in cardiotoxicity research.

Hypotheses	Novelty	Keywords	Publications, n (%)	Evaluator 1[a] score	Evaluator 2[b] score	Evaluator 3[c] score	Group consensus score
1. Development of ultra-sensitive biosensors using nanotechnology can detect early biomarkers of cardiotoxicity at extremely low concentrations	Ultra-sensitive detection of early cardiotoxicity biomarkers using nanotechnology	"ultra-sensitive biosensors," "nanotechnology," "early biomarkers," "cardiotoxicity"	0 (0)	4	4	4	4
2. Employing advanced imaging techniques such as hyperpolarized MRI[d] can enhance the sensitivity of detecting subtle cardiac changes indicative of cardiotoxicity	Enhanced sensitivity in detecting subtle cardiac changes with hyperpolarized MRI	"advanced imaging," "hyperpolarized MRI," "cardiac changes," "cardiotoxicity"	0 (0)	4	4	3	4

3. Integrating liquid biopsy techniques with next-generation sequencing can identify circulating biomarkers of cardiotoxicity with high sensitivity	High sensitivity in identifying circulating cardiotoxicity biomarkers using liquid biopsy and next-generation sequencing	“liquid biopsy,” “next-generation sequencing,” “circulating biomarkers,” “cardiotoxicity”	0 (0)	3	3	3	3
4. Utilizing single-cell transcriptomics in blood samples can detect early cardiotoxic responses by identifying rare cell populations affected by cardiotoxic agents	Early detection of cardiotoxic responses with single-cell transcriptomics in blood samples	“single-cell transcriptomics,” “blood samples,” “early cardiotoxic responses”	0 (0)	4	4	4	4

5. Developing machine learning algorithms to analyze electrocardiogram data can detect subtle and early changes in cardiac electrical activity associated with cardiotoxicity	Detection of subtle cardiac electrical activity changes with machine learning on ECG data[g]	"machine learning," "ECG data," "cardiac electrical activity," "cardiotoxicity"	0 (0)	5	5	4	5
6. Creating wearable devices with enhanced sensitivity to monitor real-time cardiac biomarkers in sweat or interstitial fluid can provide early warning signs of cardiotoxicity	Real-time monitoring of cardiac biomarkers in sweat or interstitial fluid using wearable devices	"wearable devices," "real-time monitoring," "cardiac biomarkers," "cardiotoxicity"	0 (0)	5	5	4	5
7. Applying proteomics to identify low-abundance proteins in cardiac tissue samples can improve the detection of early molecular changes	Identification of low-abundance proteins in cardiac tissues with	"proteomics," "low-abundance proteins," "cardiac tissue samples,"	0 (0)	4	4	4	4

due to cardiotoxicity	proteomics	"cardiotoxicity"					
8. Using CRISPR[e]-based detection systems to identify specific genetic markers of cardiotoxicity in patient-derived samples can increase sensitivity	Increased sensitivity in detecting genetic markers of cardiotoxicity with CRISPR-based systems	"CRISPR-based detection," "genetic markers," "patient-derived samples," "cardiotoxicity"	0 (0)	4	3	3	3
9. Developing fluorescent nanoprobes that bind to specific cardiac biomarkers can allow for real-time visualization and early detection of cardiotoxicity	Real-time visualization and early detection with fluorescent nanoprobes binding to cardiac	"fluorescent nanoprobes," "real-time visualization," "early detection," "cardiotoxicity"	0 (0)	4	4	4	4

	biomarkers						
10. Incorporating artificial intelligence with high-resolution ultrasound imaging can enhance the detection of microstructural changes in cardiac tissue indicative of early cardiotoxicity	Enhanced detection of microstructural changes in cardiac tissue using AI[f] and high-resolution ultrasound imaging	"AI," "high-resolution ultrasound imaging," "microstructural changes," "cardiotoxicity"	0 (0)	5	5	4	5
11. Leveraging metabolomics to profile metabolic changes in blood and urine samples can improve the sensitivity of detecting early cardiotoxic effects	Improved sensitivity in detecting early cardiotoxic effects with metabolomics profiling	"metabolomics," "metabolic changes," "blood and urine samples," "early cardiotoxic effects"	0 (0)	4	4	4	4

12. Employing optical coherence tomography with AI-based analysis can detect micro-level cardiac tissue changes associated with cardiotoxicity	Detection of micro-level cardiac tissue changes using OCT and AI-based analysis[h]	"optical coherence tomography," "AI-based analysis," "micro-level cardiac tissue changes," "cardiotoxicity"	0 (0)	4	4	4	4
13. Developing quantum dot-based sensors that can detect minute changes in cardiac enzyme levels associated with early cardiotoxicity	Detection of minute cardiac enzyme changes with quantum dot-based sensors	"quantum dot-based sensors," "cardiac enzyme levels," "early cardiotoxicity"	0 (0)	4	4	4	4
14. Utilizing advanced glycomics to study changes in glycosylation patterns of cardiac proteins as sensitive markers of cardiotoxicity	Sensitive markers of cardiotoxicity with advanced glycomics analysis	"advanced glycomics," "glycosylation patterns,"	0 (0)	4	4	4	4

	of glycosylation patterns	“sensitive markers,” “cardiotoxicity”					
15. Applying high-throughput screening of microRNAs in blood samples to identify sensitive early markers of cardiotoxicity	Identification of early cardiotoxic markers with high-throughput microRNA screening in blood	“high-throughput screening,” “microRNAs,” “blood samples,” “early markers,” “cardiotoxicity”	0 (0)	4	4	4	4
16. Integrating deep learning models with cardiac MRI data to enhance the sensitivity of detecting early signs of cardiotoxicity-related fibrosis	Enhanced sensitivity in detecting early signs of cardiotoxicity-related fibrosis with deep learning and cardiac MRI data	“deep learning,” “cardiac MRI data,” “early signs,” “cardiotoxicity-related fibrosis”	0 (0)	4	4	3	4

17. Using advanced electrophysiological techniques to detect subtle changes in cardiac cell electrical properties as early indicators of cardiotoxicity	Detection of subtle changes in cardiac cell electrical properties with advanced electrophysiological techniques	“advanced electrophysiological techniques,” “cardiac cell electrical properties,” “early indicators,” “cardiotoxicity”	0 (0)	4	4	3	4
18. Developing antibody-based biosensors for detecting low-abundance cardiac biomarkers in blood with high sensitivity	High sensitivity in detecting low-abundance cardiac biomarkers with antibody-based biosensors	“antibody-based biosensors,” “low-abundance biomarkers,” “blood,” “high sensitivity,” “cardiotoxicity”	0 (0)	4	4	3	3

19. Employing photoacoustic imaging combined with nanoparticle contrast agents to detect early cardiac tissue changes indicative of cardiotoxicity	Early detection of cardiac tissue changes with photoacoustic imaging and nanoparticle contrast agents	"photoacoustic imaging," "nanoparticle contrast agents," "early cardiac tissue changes," "cardiotoxicity"	0 (0)	5	5	4	5
20. Creating lab-on-a-chip devices that can perform multiplexed detection of cardiotoxicity biomarkers from a single drop of blood with high sensitivity	Multiplexed detection of cardiotoxicity biomarkers with lab-on-a-chip devices	"lab-on-a-chip devices," "multiplexed detection," "cardiotoxicity biomarkers," "high sensitivity"	0 (0)	4	4	4	4

[a]Author YL (MD and PhD, professor).

[b]Author TG (MD, final-year PhD candidate).

[c]Author CY (MD, first-year PhD student).

[d]MRI: magnetic resonance imaging.

[e]CRISPR: clustered regularly interspaced short palindromic repeats.

[f]AI: artificial intelligence.

[g]ECG: electrocardiogram

[h]OCT: optical coherence tomography
