# Supplementary material for: AI-Assisted Hypothesis Generation to Address Challenges in Cardiotoxicity Research: Simulation Study Using ChatGPT With GPT-4o
Source: J Med Internet Res. 2025 May 15;27:e66161. doi: 10.2196/66161 (PMC12123237; doi:10.2196/66161)
Supplement: Multimedia Appendix 4 [file jmir_v27i1e66161_app4.pdf]

Multimedia Appendix 4. Evaluation of hypotheses to overcome the challenge of the lack of reliable biomarkers in cardiotoxicity research.

Hypotheses	Novelty	Keywords	Publications (n=54), n (%)	Evaluator 1[a] score	Evaluator 2[b] score	Evaluator 3[c] score	Group consensus score
1. Utilizing multi-omics approaches (genomics, proteomics, metabolomics) to identify novel and reliable biomarkers for cardiotoxicity	Integration of multi-omics data for comprehensive biomarker discovery	"multi-omics," "genomics," "proteomics," "metabolomics," "biomarkers," "cardiotoxicity"	0 (0)	4	4	4	4
2. Developing CRISPR[d]-based screening methods to discover genes and pathways involved in cardiotoxicity, leading to the identification of new biomarkers	CRISPR-based gene and pathway discovery for new cardiotoxicity biomarkers	"CRISPR," "gene discovery," "pathway discovery," "biomarkers," "cardiotoxicity"	0 (0)	5	4	3	4

3. Applying single-cell RNA sequencing to cardiac tissues to uncover specific gene expression profiles that serve as reliable biomarkers for cardiotoxicity	Single-cell RNA sequencing to identify specific gene expression profiles	"single-cell RNA sequencing," "gene expression," "biomarkers," "cardiotoxicity"	1 (2)	4	3	4	3
4. Investigating the role of non-coding RNAs (microRNAs, long non-coding RNAs) in cardiotoxicity to identify them as potential biomarkers	Role of non-coding RNAs as novel cardiotoxicity biomarkers	"non-coding RNAs," "microRNAs," "long non-coding RNAs," "biomarkers," "cardiotoxicity"	1 (2)	4	3	4	3
5. Conducting large-scale genome-wide association studies to find genetic variants associated with increased risk of cardiotoxicity, which	Large-scale GWAS to find genetic variants linked to cardiotoxicity risk[f]	"GWAS," "genetic variants," "cardiotoxicity	1 (2)	4	4	4	4

can be used as biomarkers.		risk,” “biomarkers”					
6. Using high-throughput proteomic screening of blood samples from patients treated with cardiotoxic drugs to identify protein biomarkers indicative of cardiotoxicity	High-throughput proteomics for identifying protein biomarkers in blood	“high-throughput proteomics,” “blood samples,” “protein biomarkers,” “cardiotoxicity”	0 (0)	4	4	4	4
7. Developing metabolomic profiles of patients undergoing treatment with cardiotoxic agents to identify metabolic biomarkers that predict cardiotoxicity	Metabolomic profiling to discover metabolic biomarkers	“metabolomics,” “metabolic profiling,” “biomarkers,” “cardiotoxicity”	50 (93)	3	2	4	2

8. Investigating extracellular vesicles (exosomes) released by cardiomyocytes under stress to identify their molecular cargo as biomarkers for cardiotoxicity	Identification of molecular cargo in exosomes as biomarkers	“extracellular vesicles,” “exosomes,” “molecular cargo,” “biomarkers,” “cardiotoxicity”	0 (0)	4	4	3	4
9. Applying advanced glycomics to study changes in glycosylation patterns of cardiac proteins as biomarkers of cardiotoxicity	Advanced glycomics for changes in glycosylation patterns	“advanced glycomics,” “glycosylation patterns,” “biomarkers,” “cardiotoxicity”	0 (0)	4	4	4	4
10. Utilizing artificial intelligence and machine learning to analyze large datasets from clinical trials to identify novel biomarkers for cardiotoxicity	AI[e] and machine learning to analyze large clinical datasets	“AI,” “machine learning,” “clinical datasets,”	0 (0)	4	4	3	4

		"biomarkers," "cardiotoxicity"					
11. Exploring the gut-heart axis to identify microbiota-derived metabolites that serve as biomarkers for cardiotoxicity	Microbiota-derived metabolites as biomarkers for cardiotoxicity	"gut-heart axis," "microbiota-derived metabolites," "biomarkers," "cardiotoxicity"	0 (0)	4	4	4	4
12. Using integrative bioinformatics approaches to combine data from different omics studies to pinpoint robust biomarkers for cardiotoxicity	Integrative bioinformatics for combining multi-omics data	"integrative bioinformatics," "multi-omics data," "biomarkers," "cardiotoxicity"	0 (0)	4	4	4	4

13. Investigating the role of immune system activation and inflammation in cardiotoxicity to identify cytokines and other immune markers as reliable biomarkers	Immune system activation and inflammation markers as biomarkers	“immune system activation,” “inflammation markers,” “biomarkers,” “cardiotoxicity”	1 (2)	4	4	3	4
14. Developing high-sensitivity assays to detect post-translational modifications in cardiac proteins as early biomarkers of cardiotoxicity	High-sensitivity assays for post-translational modifications	“high-sensitivity assays,” “post-translational modifications,” “biomarkers,” “cardiotoxicity”	0 (0)	4	4	4	4
15. Applying lipidomics to profile changes in lipid composition in blood samples as biomarkers for cardiotoxicity	Lipidomics for profiling changes in lipid composition	“lipidomics,” “lipid composition,” “biomarkers,” “cardiotoxicity”	0 (0)	4	4	4	4

16. Using longitudinal studies with repeated sampling to track changes in potential biomarkers over time in patients treated with cardiotoxic drugs	Longitudinal studies for tracking biomarker changes over time	"longitudinal studies," "repeated sampling," "biomarkers," "cardiotoxicity"	0 (0)	4	4	4	4
17. Investigating the epigenetic changes in cardiomyocytes exposed to cardiotoxic agents to identify DNA methylation or histone modification patterns as biomarkers	Epigenetic changes as biomarkers for cardiotoxicity	"epigenetic changes," "DNA methylation," "histone modifications, biomarkers," "cardiotoxicity"	0 (0)	4	4	4	4
18. Developing high-throughput screening assays to identify oxidative stress markers in cardiomyocytes as biomarkers for cardiotoxicity	High-throughput assays for oxidative stress markers	"high-throughput screening," "oxidative stress markers,"	0 (0)	5	5	3	5

		"biomarkers," "cardiotoxicity"					
19. Exploring the role of cell-free DNA and RNA in the blood as non-invasive biomarkers for early detection of cardiotoxicity	Cell-free DNA and RNA as non-invasive biomarkers	"cell-free DNA," "cell-free RNA," "non-invasive biomarkers," "cardiotoxicity"	0 (0)	4	4	4	4
20. Utilizing 3D bioprinted cardiac tissue models to study molecular changes under drug exposure and identify reliable biomarkers for cardiotoxicity	3D bioprinted models for studying molecular changes	"3D bioprinted models," "molecular changes," "biomarkers," "cardiotoxicity"	0 (0)	5	5	4	5

[a]Author YL (MD and PhD, professor).

[b]Author TG (MD, final-year PhD candidate).

[c]Author CY (MD, first-year PhD student).

[d]CRiSPR: clustered regularly interspaced short palindromic repeats.

[e]AI: artificial intelligence.

[f]GWAS: genome-wide association study.
