# Supplementary material for: AI-Assisted Hypothesis Generation to Address Challenges in Cardiotoxicity Research: Simulation Study Using ChatGPT With GPT-4o
Source: J Med Internet Res. 2025 May 15;27:e66161. doi: 10.2196/66161 (PMC12123237; doi:10.2196/66161)
Supplement: Multimedia Appendix 5 [file jmir_v27i1e66161_app5.pdf]

Multimedia Appendix 5. Evaluation of hypotheses to overcome the challenge of the limitations of animal models in cardiotoxicity research.

Hypotheses	Novelty	Keywords	Publications (n=72), n (%)	Evaluator 1[a] score	Evaluator 2[b] score	Evaluator 3[c] score	Group consensus score
1. Developing human induced pluripotent stem cell -derived cardiomyocyte models to better mimic human cardiac responses to cardiotoxic agents	iPSC-derived cardiomyocyte models for human-like responses[f]	"iPSC-derived cardiomyocytes," "human cardiac responses," "cardiotoxicity"	28 (39)	5	5	5	5
2. Utilizing 3D bioprinted human heart tissues that replicate the structural and functional characteristics of human hearts for cardiotoxicity testing	3D bioprinted heart tissues replicating human heart characteristics	"3D bioprinted heart tissues," "structural and functional characteristics," "cardiotoxicity"	0 (0)	5	5	5	5

3. Employing organ-on-a-chip technology, specifically heart-on-a-chip systems, to create more accurate and human-relevant models for cardiotoxicity studies	Heart-on-a-chip systems for accurate human-relevant models	"organ-on-a-chip," "heart-on-a-chip," "accurate models," "cardiotoxicity"	1 (1)	4	4	4	4
4. Applying CRISPR[d]-Cas9 gene editing to create patient-specific iPSC-derived cardiomyocytes for personalized cardiotoxicity testing	CRISPR-Cas9 for patient-specific cardiomyocyte models	"CRISPR-Cas9," "patient-specific cardiomyocytes," "personalized testing," "cardiotoxicity"	0 (0)	4	4	3	4
5. Using human cardiac organoids to study cardiotoxic responses in a controlled, three-dimensional environment that closely mimics human heart tissue	Human cardiac organoids in a controlled, 3D environment	"human cardiac organoids," "3D environment," "cardiotoxic responses"	0 (0)	4	4	4	4

6. Integrating multi-organ-on-a-chip systems to study the systemic effects of cardiotoxic agents and their interactions with other organs	Multi-organ-on-a-chip systems for systemic effects study	“multi-organ-on-a-chip,” “systemic effects,” “cardiotoxicity”	0 (0)	4	3	4	3
7. Developing genetically modified animal models that express human cardiac-specific genes to improve the relevance of cardiotoxicity studies	Genetically modified animal models expressing human cardiac genes	“genetically modified animal models,” “human cardiac genes,” “relevance,” “cardiotoxicity”	3 (4)	4	4	3	3
8. Employing advanced computational modeling and simulations to predict cardiotoxicity based on human cardiac cell data	Computational modeling and simulations with human cardiac data	“computational modeling,” “simulations,” “human cardiac data,” “cardiotoxicity”	35 (49)	4	2	3	2

9. Using human explant heart tissues in ex vivo studies to directly observe human-specific cardiotoxic responses	Human explant heart tissues for direct human-specific responses	“human explant heart tissues,” “ex vivo studies,” “human-specific responses,” “cardiotoxicity”	0 (0)	5	5	3	4
10. Creating chimeric animal models with humanized hearts to better replicate human cardiotoxicity in vivo	Chimeric animals with humanized hearts for better replication	“chimeric animals,” “humanized hearts,” “replication,” “cardiotoxicity”	0 (0)	4	4	3	4
11. Implementing single-cell transcriptomics and proteomics on human cardiac tissues to identify specific biomarkers and pathways involved in cardiotoxicity, which can	Single-cell transcriptomics and proteomics for biomarkers and pathways	“single-cell transcriptomics,” “proteomics,” “biomarkers,” “pathways,”	0 (0)	4	4	4	4

be used to refine in vitro models		"cardiotoxicity"					
12. Utilizing human cardiac microtissues engineered with diverse cell types to study the complex interactions and cardiotoxic effects in a more representative model	Human cardiac microtissues with diverse cell types for complex interactions	"human cardiac microtissues," "diverse cell types," "complex interactions," "cardiotoxicity"	0 (0)	4	4	4	4
13. Developing patient-derived xenografts models for cardiotoxicity testing, where human heart tissues are implanted in immunodeficient mice to study human-specific drug responses	PDX models with human heart tissues[g]	"patient-derived xenografts," "PDX models," "human heart tissues," "drug responses,"	0 (0)	4	4	4	4

		"cardiotoxicity"					
14. Using humanized zebrafish models with human cardiac genes to study the cardiotoxic effects of drugs in a high-throughput manner	Humanized zebrafish models with human cardiac genes for high-throughput studies	"humanized zebrafish models," "human cardiac genes," "high-throughput ," "cardiotoxicity"	0 (0)	4	4	3	4
15. Applying human cardiac spheroid models to evaluate the cumulative effects of chronic exposure to cardiotoxic agents in a three-dimensional context	Human cardiac spheroid models for chronic exposure studies in 3D context	"human cardiac spheroids," "chronic exposure," "3D context,"	0 (0)	4	4	4	4

		"cardiotoxicity"					
16. Implementing AI[e]-driven analysis of human cardiac cell responses to cardiotoxic agents to refine in vitro models based on real-world data	AI-driven analysis for refining in vitro models with real-world data	"AI-driven analysis," "human cardiac cells," "in vitro models," "real-world data," "cardiotoxicity"	0 (0)	4	4	4	4
17. Developing lab-on-a-chip devices that incorporate human cardiac cells and simulate the mechanical forces experienced by the heart to study drug-induced cardiotoxicity	Lab-on-a-chip devices simulating mechanical forces on human cardiac cells	"lab-on-a-chip," "mechanical forces," "human cardiac cells," "cardiotoxicity"	2 (3)	5	4	4	4

18. Using human cardiac tissue slices in vitro to assess the electrophysiological and contractile responses to cardiotoxic agents	Human cardiac tissue slices for electrophysiological and contractile responses	“human cardiac tissue slices,” “electrophysiological responses,” “contractile responses,” “cardiotoxicity”	1 (1)	5	5	4	5
19. Integrating human-specific metabolic and genetic profiles into in vitro cardiotoxicity models to improve their predictive accuracy	Integrating human-specific metabolic and genetic profiles in in vitro models	“human-specific metabolic profiles,” “genetic profiles,” “in vitro models,” “cardiotoxicity”	0 (0)	4	4	4	4

20. Employing high-throughput screening platforms with human cardiac cells to identify and validate new cardiotoxicity biomarkers and therapeutic targets	High-throughput screening with human cardiac cells for biomarker discovery	"high-throughput screening," "human cardiac cells," "biomarkers," "therapeutic targets," "cardiotoxicity"	2 (3)	4	4	4	4

[a]Author YL (MD and PhD, professor).

[b]Author TG (MD, final-year PhD candidate).

[c]Author CY (MD, first-year PhD student).

[d]CRiSPR: clustered regularly interspaced short palindromic repeats.

[e]AI: artificial intelligence.

[f]iPSC: induced pluripotent stem cell

[g]PDX: patient-derived xenograft
